# Morphological redescription and genetic characterization of the enigmatic *Nannopus palustris* Brady, 1880 (Copepoda, Harpacticoida, Nannopodidae)

**DOI:** 10.3897/zookeys.1278.188797

**Published:** 2026-04-27

**Authors:** Lesya Garlitska, Elena Chertoprud, Tatyana Mayor, Maria Saburova, Gor Gevorgyan, Andrey Azovsky

**Affiliations:** 1 Scientific Center of Zoology and Hydroecology, 7 P. Sevak Str., Yerevan, 0014, Armenia Scientific Center of Zoology and Hydroecology Yerevan Armenia; 2 A.N. Severtsov Institute of Ecology and Evolution, RAS, 33 Leninsky Prospekt, Moscow, 119071, Russia Lomonosov Moscow State University Moscow Russia; 3 Limnological Institute SB RAS, 3 Ulan-Batorskaya Str., Irkutsk, 664033, Russia Limnological Institute SB RAS Irkutsk Russia; 4 Coastal and Marine Resources Program, Environment and Life Sciences Research Center, Kuwait Institute for Scientific Research, PO Box 1638, Salmiya, 22017, Kuwait A.N. Severtsov Institute of Ecology and Evolution Moscow Russia; 5 Lomonosov Moscow State University, 1/12 Leninskie Gory, Moscow, 119991, Russia Kuwait Institute for Scientific Research Salmiya Kuwait

**Keywords:** Harpacticoida, molecular genetics, morphology, neotype, taxonomy

## Abstract

*Nannopus palustris* Brady, 1880 is considered to be distributed worldwide and is the type species of *Nannopus* Brady, 1880 that, in turn, is the type genus of the family Nannopodidae Brady, 1880. However, its original description and subsequent amendments are discrepant and is insufficiently detailed, giving rise to considerable taxonomic confusion thus hampering species delimitation, further revisions, and other taxonomical acts. This paper presents a detailed morphological redescription of both sexes of *N. palustris* with the designation of a neotype from material recently collected from the type locality. We compared key characteristics of the newly collected specimens of *N. palustris* with those of other species of *Nannopus*. Morphological and molecular analyses, including 18S rDNA, support the subdivision of the genus into two species groups as proposed by Sciberras et al. (2021). Specimens from several distant populations, previously identified as *N. palustris* were reexamined. Specimens from the Westerschelde estuary (SW Netherlands) and Small Adzhalyk Estuary (NW Black Sea) were found to be identical to those from the type locality and were attributed to *N. palustris*. Specimens from the White Sea, however, turned out to be conspeciﬁc with *N. procerus* Fiers & Kotwicki, 2013. Specimens from the Vellar estuary (Bay of Bengal) and Lake Hinuma (Japan) likely represent distinct, yet undescribed species of *Nannopus*. An updated generic diagnosis is provided, offering a clearer framework for future taxonomic studies.

## Introduction

*Nannopus palustris* Brady, 1880 (Harpacticoida: Nannopodidae) is a striking example of a taxon with a complex and bewildering history. Brady (1880) erected the genus *Nannopus* Brady, 1880 for a new species, *N. palustris*, which was collected from brackish water pools at Seaton Sluice, Northumberland, Great Britain. Originally, Brady (1880) established *Nannopus* as the type genus of a new subfamily, Nannopinae Brady, 1880, within the family Harpacticidae Dana, 1846. Later, Sars (1909) assigned *Nannopus* to Cletodidae Scott T., 1904, and Por (1986) transferred it to Huntemanniidae Por, 1986. Huys (2009) synonymized Huntemanniidae with Nannopinae, corrected the spelling of the family name to Nannopodidae, and fixed the genus *Nannopus* as the type for the family.

However, the outright vagueness of Brady’s (1880) description, which was based on a single or possibly two specimens, with insufﬁcient and entangled illustrations, gave rise to considerable taxonomic confusion on the reinterpretation of the species boundaries. Subsequent descriptions based on specimens claimed to represent *N. palustris* (e.g., Canu 1892; Scott 1902; Sars 1909; Gurney 1932; etc.) differed in many details from the original text and illustrations of Brady. Later, this species was repeatedly reported worldwide, from the Arctic to the tropics, and in different habitats from marine to freshwater situations (see distribution map in Garlitska et al. 2012). Several authors also provided some morphological details of the specimens examined and noted particular discrepancies between their observations and earlier descriptions (e.g., Willey 1923, 1929; Monchenko and Polishchuk 1969; Wells 1971; Coull and Fleeger 1977; Letova 1982; Staton et al. 2005). For these reasons, *N. palustris* has long been assumed to be a single, cosmopolitan, and eurytopic but highly variable species (Wells 1971).

However, comparative morphological and molecular analyses of several sympatric “morphs”, and geographically distant populations attributed to *N. palustris*, have certainly revealed that it is not one polymorphic species but a complex of pseudocryptic species, i.e., genetically and morphologically divergent lineages (Staton et al. 2005; Garlitska et al. 2012). During the next decade, a number of new species were described (including nine from Korean waters), doubling the total richness to 20 species. Concurrently, the genus as a whole was subjected to reconsideration. Fiers and Kotwicki (2013) postulated that *Nannopus* can be separated into two different lineages. Kim et al. (2017) supported this suggestion and, starting from Brady’s original generic diagnosis, reinstated the genus *Iliophilus* Lilljeborg, 1902 to accommodate all the species with a 2-segmented P3 endopod, including the *N. palustris* sensu Canu, 1892, as a new species, *I. canui* Kim, Choi & Yoon, 2017. However, Vakati and Lee (2017) doubted this proposal. Then, Vakati et al. (2019), on the basis of both morphological analysis and molecular data, argued quite forcefully against the validity of this generic separation. Recently, Sciberras et al. (2021) agreed with Vakati et al. (2019) and maintained *Nannopus* and *Iliophilus* as synonyms and subdivided the former into two species groups based on the presence or absence of an inner seta on P1exp-2 but set *N. palustris* s. str. apart from the others and omitted it from their key to the species of *Nannopus*.

Thus, one of the primary obstacles to further progress in disentangling this species complex lies in the inadequacy of existing descriptions of the type species *N. palustris*. Hence, Fiers and Kotwicki (2013), Vakati et al. (2019) and Sciberras et al. (2021), stressed the need for a redescription of this problematic species based on topotypic material, with the eventual designation of a neotype, to avoid any taxonomic confusion. Brady’s (1880) original description was based on a single or possibly two females, but no holotype was formally fixed. Unfortunately, examination of the Brady collections held at the Natural History Museum, London, proved that the original material was poorly preserved, and its reassessment was not possible. Moreover, a recent request revealed that the only specimen of *N. palustris* preserved in a single slide in the NHML collection (catalog number 1951.8.10.758) comes from another locality and is not marked as part of the type series (Miranda Lowe, Principal Curator (Crustacea & Cnidaria collections), Natural History Museum, London, pers. comm. 20^th^ February 2026). Thus, the type material is unavailable for re-analysis and is most probably no longer extant. Therefore, based on the exceptional need of a name-bearing type of the species to define the nominal taxon objectively, and to clarify its taxonomic status, we have designated a neotype from among the new specimens collected from the type locality, in accordance with ICZN article 75. We also present a redescription of *N. palustris* s. str. based on newly collected specimens from the type locality, providing evidence that the neotype is essentially consistent with the original description, and indicate the characters that differentiate this species from other congeners. Hence the neotype designation meets the qualifying criteria listed in ICZN Art 75.3.

## Materials and methods

### Sampling

**Figure 1.**
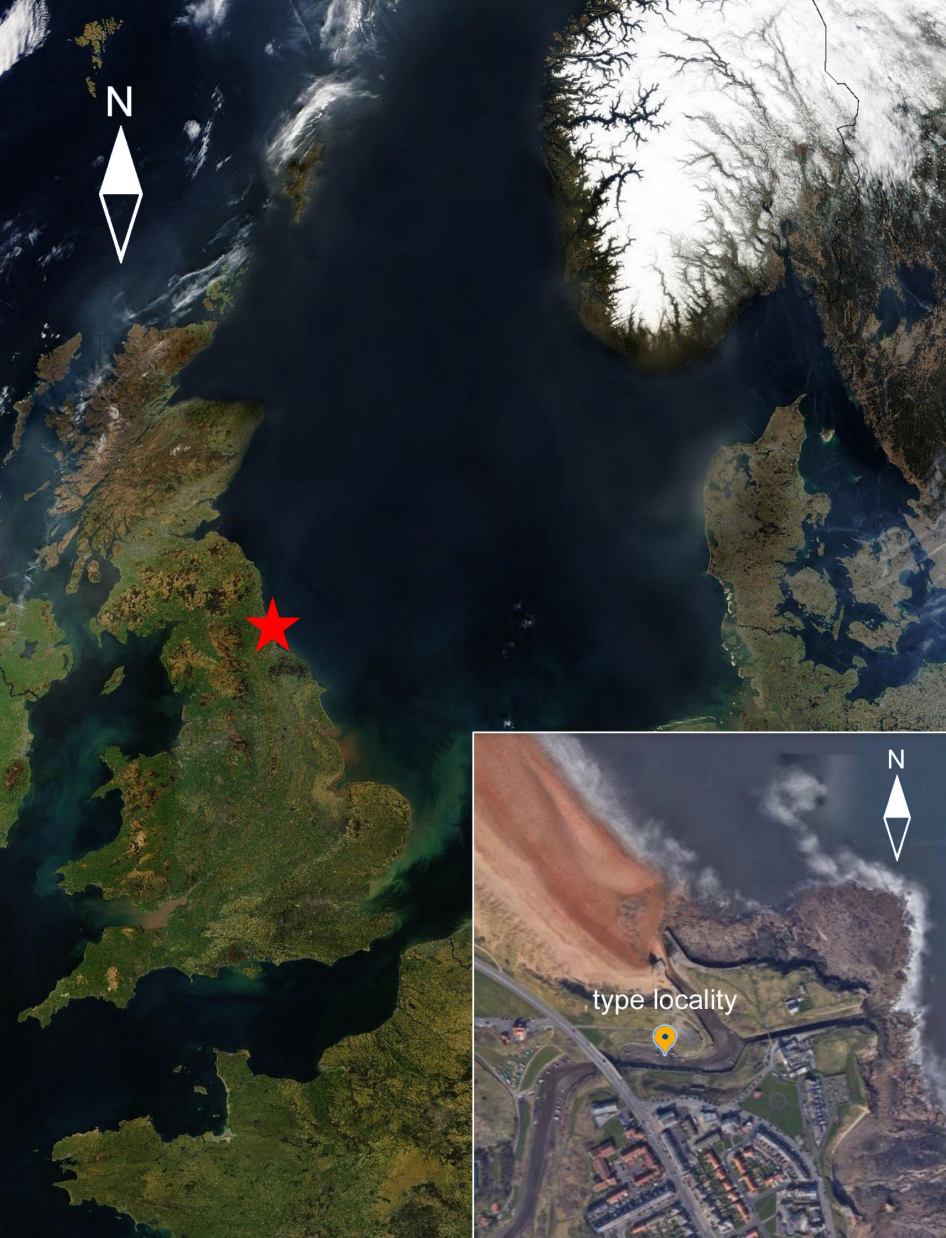
Type locality of *Nannopus palustris* Brady, 1880. Map data 2026 (C) Google https://maps.app.goo.gl/vND97nxjPbifSAcf7 & by MODIS Rapid Response Team, NASA/GSFC – Source: http://visibleearth.nasa.gov/view_rec.php?id=5341 first upload: 7 April 2004, Wikipedia by user: Southpark. This is a modified version of https://commons.wikimedia.org/w/index.php?curid=552792.

The study area was the salt marsh at Seaton Sluice, Northumberland, UK (55°05.06'N, 01°28.42'E), indicated as the type locality of *N. palustris* (Brady, 1880) (Fig. 1). The specimens were qualitatively sampled in August 2013 and September 2023 at low tide by collecting mud manually above the anoxic layer (the upper ~ 0.5 cm of sediments) from an area ≥ 5 m^2^. In all our samples from Seaton Sluice, the *Nannopus* specimens redescribed here were the only Nannopodidae and, according to a number of features, corresponded to original description; thus, it is unlikely that they belonged to a different species than the specimens described by Brady (1880).

### Additional material examined in the present study

Netherlands • 69 specimens preserved in 96% ethanol; North Sea, Westerschelde estuary, Paulina intertidal ﬂat; 51°21'N, 3°43'E; October 2011; leg. M. De Troch.

Ukraine • 23 specimens preserved in 96% ethanol; NW Black Sea, Small Adzhalyk Estuary; 46°37'N, 31°1'E; August 2003 and July 2011; leg. L. Garlitska.

Russian Federation • ca. 100 specimens preserved in 96% ethanol; White Sea, Kandalaksha Gulf, intertidal sandflat in Chernaya Bight; 66°31.25'N, 32°58.72'E; 2011–2020 yrs; leg. E. Chertoprud and L. Garlitska.

India • 2 ♀ and 2 ♂ dissected on 4 slides each, 5 specimens preserved in formaldehyde; Bay of Bengal, intertidal beach in Vellar estuary, Parangipettai (Porto Novo); 11°29'N, 79°47'E; April 2012; leg. L. Garlitska.

Additional material forms part of the first and second authors’ personal collections.

### Morphological examination and taxonomic identification

In the laboratory, the sediments were washed with tap water, passed through a 40-µm sieve, and then preserved in 96% ethanol. The copepods were picked out and sorted under a stereomicroscope (MZ APO, Leica Microsystems GmbH, Wetzlar, Germany).

The habitus was photographed, and body length measurements were made from whole specimens mounted in glycerin. The harpacticoids were dissected in glycerin, their parts were mounted individually in lactophenol, and the slides were sealed with transparent nail varnish. Observations were made with a stereomicroscope (MZ APO, Leica Microsystems GmbH, Wetzlar, Germany) and a light microscope (CX41, Olympus Corporation, Tokyo, Japan). All the drawings were made with a drawing tube attached to the light microscope (CX41, Olympus Corporation, Tokyo, Japan). The morphological terminology followed that of Huys and Boxshall (1991).

**A1** antennule;

**A2** antenna;

**aes** aesthetasc;

**cphth** cephalothorax;

**CR** caudal ramus/rami;

**enp-1-3** first-third segment of endopod;

**exp-1-3** first-third segment of exopod;

**GDS** genital double-somite;

**md** mandible;

**mx** maxilla;

**mxl** maxillule;

**mxp** maxilliped;

**P1-P6** swimming legs 1–6.

Several specimens were examined in a scanning electron microscope (SEM). The specimens were fixed with 4% formaldehyde, cleaned with a Branson B200 ultrasonic cleaner, dehydrated through graded ethanol solutions, dried using a critical point dryer EM CPD300 (Leica Microsystems GmbH, Wetzlar, Germany), mounted on stubs, and sputter-coated with gold-palladium in a vacuum sputter coater JFC-1600 (Jeol Ltd., Japan). Observations were conducted using a Vega3 scanning electron microscope (TESCAN Brno, Czech Republic) operated at 10 kV. Digital photographs were processed and combined into plates using Adobe Photoshop CS4.

### DNA extraction, amplification, and sequencing

The specimens for molecular analyses were preserved in 96% ethanol. Total DNA was isolated from the somatic tissue (cephalothorax and abdomen) of three specimens of *N. palustris* whose tissue was digested for 1–3 hours at 56 °C in a lysis solution containing 2× 10× Tersus Plus buffer (exluding Mg^2+^) (Evrogen, Moscow, Russia) and 0.1 mg/ml proteinase K, followed by enzyme inactivation at 96 °C for 5 minutes. Genetic analysis focused on two markers: mitochondrial cytochrome c oxidase subunit I (COI) and 18S rRNA genes (18S). Amplification was performed in a 10 µl reaction mixture containing 5–10 ng of total DNA, 1× Tersus Plus buffer, 3.5 mM MgCl2, 0.5 µM of each primer, 0.2 mM of each dNTP, and 0.5 units of Tersus DNA polymerase (Evrogen, Russia). Universal primers for COI and 18S amplification were used (Spears et al. 1992; Geller et al. 2013; Leray et al. 2013). The thermal profile for 18S amplification consisted of initial denaturation at 95 °C for 4 min; 35–40 cycles of 94 °C for 15 sec, 50 °C for 20 sec, and 72 °C for 1 min; and a final extension at 72 °C for 4 min. The thermal cycling profile described by Leray et al. (2013) was employed to amplify the COI. PCR products were separated by electrophoresis on a 0.6% agarose gel using 0.5× TA buffer. Bands containing the target amplicons were excised from the gel. These gel fragments were subjected to a freeze–thaw purification method: they were frozen overnight at -20 °C, thawed, and then centrifuged for 10 min at 10,000 rpm. The resulting supernatant was used as the template for sequencing. Sanger sequencing was performed directly using the ABI PRISM BigDye Terminator v. 3.1 kit (Thermo Fisher Scientific, Waltham, USA) on a Nanophor 05 genetic analyzer (Sintol, Moscow, Russia).

### Phylogenetic analysis

Chromatograms were examined manually using UNIPRO UGENE v. 49.1 (Okonechnikov et al. 2012). We obtained and deposited three COI sequences (275–303 bp; accessions PX936547, PX936548, PX980509) and three 18S sequences (399–566 bp; accessions PX936533–PX936535) in the GenBank database of the National Center for Biotechnology Information (NCBI). Our newly obtained sequences were combined with additional sequences of *Nannopus* and other harpacticoids retrieved from GenBank (accession numbers are listed in Tables 1, 2) for phylogenetic analyses. Sequence alignments were performed with the Pairwise Deletion option in MEGA X (Kumar et al. 2018), which was also used to compute uncorrected *p*-distances. Partitioned analysis for multi-gene alignments was performed with IQ-TREE3 (Chernomor et al. 2016; Wong et al. 2025). For *N. palustris*, where sequences from three individuals were identical for both markers, we selected the COI and 18S sequences from specimen 25-12 (PX936535, PX936548), as they comprised the longest available 18S read. For the remaining species of *Nannopus*, we used sequences for both markers that originated from the same individuals, as available in GenBank. The 18S alignment (1843 bp) was manually concatenated with the COI alignment (675 bp) for each individual. The best-fit nucleotide substitution models were selected using ModelFinder with -m MFP+MERGE option (integrated in IQ-TREE3) under the Bayesian Information Criterion (BIC) (Kalyaanamoorthy et al. 2017). The best-fit partition scheme used separate substitution models: TIM2+I for 18S and TIM+F+R3 for COI. Branch support was evaluated using standard nonparametric bootstrapping and SH-aLRT test. Final trees were visualized and annotated in Interactive Tree Of Life (iTOL) v.6.8.1 (Letunic and Bork 2021).

**Table 1.** Characteristics of newly generated sequences of *Nannopus palustris* used in this study.

Gene	Specimen ID	Sequence length (bp)	GenBank accession	Sequencing direction	Sequence reliability
COI	25_12	290	PX936548	Single-stand	High-quality read; manually verified; no ambiguous sites
COI	25_13	303	PX980509	Single-stand	High-quality read; manually verified; no ambiguous sites
COI	25_16	275	PX936547	Single-stand	High-quality read; manually verified; no ambiguous sites
18S	25_12	566	PX936535	Single-stand	Clear chromatogram; manually inspected; no ambiguous sites
18S	25_13	399	PX936533	Single-stand	Moderate quality; manually inspected; no ambiguous sites
18S	25_16	546	PX936534	Single-stand	Clear chromatogram; manually inspected; no ambiguous sites

**Table 2.** The *p*-distances (in %) between species of *Nannopus* from 18S sequences (supra-diagonal elements) and from COI sequences (sub-diagonal elements). NCBI accession numbers of sequences obtained in this study are shown in bold.

Sequence #	NCBI ID	Species	Sequence #										
			1	2	3	4	5	6	7	8	9	10	11
1	**PX936548**	*N. palustris*		0	0	6.7	5.1	6.2	5.1	1.8	2.1	1.3	1.3
2	**PX980509**	*N. palustris*	0		0	6.7	5.1	6.2	5.1	1.8	2.1	1.3	1.3
3	**PX936547**	*N. palustris*	0	0		6.7	5.1	6.2	5.1	1.8	2.1	1.3	1.3
4	MK051351.1	*Nannopus* sp. 6	25.9	25.9	25.9		5.4	2.3	5.4	6.2	5.1	5.6	5.6
5	MK051299.1	*N. minutus*	24.3	24.3	24.3	24.7		4.9	0	5.6	5.6	5.1	5.1
6	MK051362.1	*N. dimorphicus*	24.7	24.7	24.7	26.7	30		4.9	5.9	4.9	5.4	5.4
7	MK051236.1	*N. ganghwaensis*	23.9	23.9	23.9	21	27.6	25.5		5.6	5.6	5.1	5.1
8	MK051399.1	*N. parvipilis*	26.7	26.7	26.7	30	27.2	28.8	31.3		1.3	0.5	0.5
9	MK051322.1	*N. bulbiseta*	26.3	26.3	26.3	25.5	24.3	24.3	22.6	24.7		0.8	0.8
10	MK051307.1	*N. parvus*	25.5	25.5	25.5	30	25.9	25.9	28.4	25.5	19.8		0
11	MK051290.1	*N. serratus*	32.1	32.1	32.1	32.5	30.9	27.2	29.6	22.2	24.3	26.3	

### Institutional abbreviation

The neotype and other specimens studied are housed in the following institution:

**SCZHE** Scientific Center of Zoology and Hydroecology, Yerevan, Republic of Armenia.

## Results

### Phylum Arthropoda Gravenhorst, 1843

**Subphylum Crustacea Brünnich, 1772**

**Superclass Multicrustacea Regier et al., 2010**

**Class Copepoda Milne Edwards, 1840**

**Order Harpacticoida Sars G.O., 1903**

**Family Nannopodidae Brady, 1880**

**Subfamily Nannopinae Brady, 1880**

#### Genus Nannopus

Taxon classificationAnimaliaHarpacticoidaNannopodidae

Brady, 1880

44CB08A8-AF90-5080-951D-93D0BC93B796

##### Generic diagnosis

(amended from Fiers and Kotwicki 2013; Kim et al. 2017; Vakati et al. 2016, 2019; Vakati and Lee 2017, 2021; Sciberras et al. 2021). Nannopodidae. Body fusiform, slightly dorsoventrally flattened. Body ornamentation consisting of dorsal denticles and horizontal rows of setules and sensilla. Rostrum bell-shaped, fused to cephalothorax; anterior margin furnished with setules and one pair of sensilla. Cephalothorax anteriorly attenuated in dorsal view; posterior margin serrate, with several sensilla. Prosome 4-segmented, comprising cephalothorax and three subequal pedigerous somites. The female genital and first abdominal somite fused, forming a genital double-somite. Second abdominal somite with row of spinules on the ventral posterior margin. Operculum well developed, with rows of setules. Antennule 5-segmented, short in female; 2^nd^ segment with tri-articulate seta inserted dorsally; 3^rd^ segment with aesthetasc, and 5^th^ with acrothek. Male antennule 5–6-segmented, chirocer. Antenna with one or two abexopodal setae on allobasis; 1-segmented exopod with three or four setae; endopod with six or seven setae. Mandibular palp 1-segmented, broad, with 3–5 elements. Maxillule praecoxal arthrite with nine or ten elements at the distal margin. Cephalothorax with/without integumental windows. P1–P4 exopods 3-segmented; P1–P4 endopods 1-2:2:1-2:1-segmented. P2–P3exp-3 with 2-3–2-3 (♀): 2-4–2-4 (♂) outer spines. P3enp-2 of male present or absent. P4exp-3 inner subdistal element setiform or pectinate. Female P5 baseoendopod plate-like, not developed, with three or four setae; exopod with four or five setae. Female P6 represented by 1 seta. P6 articulation of males asymmetrically or symmetrically confluent; represented by two or three setae. Female with two egg-sacs, male with one or two spermatophores. Caudal rami cylindrical or trapezoidal, clearly separated from anal somite; with 7 setae; seta V well developed and longest.

##### Type species.

*Nannopus palustris* Brady, 1880 (by monotypy).

##### Other species.

*N. bulbiseta* Vakati & W. Lee, 2017, *N. canui* Sciberras, Cazzaniga & Huys, 2021, *N. cylindricus* Vakati & Lee, 2021, *N. didelphis* Fiers & Kotwicki, 2013, *N. dimorphicus* Vakati & W. Lee, 2017, *N. flexibilis* (Lilljeborg, 1902), *N. ganghwaensis* Vakati, Kihara & W. Lee, 2017, *N. hirsutus* Fiers & Kotwicki, 2013, *N. minutus* Vakati & W. Lee, 2017, *N. parvipilis* Kim, J.G., Choi & Yoon, 2017, *N. parvus* Vakati & W. Lee, 2017, *N. perplexus* (Sars G.O., 1909), *N. procerus* Fiers & Kotwicki, 2013, *N. robustus* Vakati & Lee, 2021, *N. scaldicola* Fiers & Kotwicki, 2013, *N. serratus* Vakati & W. Lee, 2017, *N. sinusalbi* Sciberras, Cazzaniga & Huys, 2021, *N. unisegmentatus* Shen & Tai, 1964; subspecies *N. palustris tiberiadis* Por, 1968.

#### Nannopus palustris

Taxon classificationAnimaliaHarpacticoidaNannopodidae

Brady, 1880

CD3E47CF-EE2F-5063-9AC2-68549EC3CFD5

Figs 2, 3, 4, 5, 6, 7, 8, 9

##### Synonymy.

*Nannopus palustris* sensu T. Scott 1902.

##### Type locality.

Seaton Sluice, Northumberland, UK (55°05.06'N, 01°28.42'E).

##### Material examined.

• 1 ♀ designated as the neotype, dissected on 4 slides, deposited at the Zoological Museum of SCZHE, reg. no. SCZHE-COP-1–4; • 1 ♀ undissected on 1 slide, same collection data, reg. no. SCZHE-COP-5; • 1 ♂ dissected on 4 slides, same collection data, reg. no. SCZHE-COP-6–9; • 1 ♂ undissected on 1 slide, same collection data, reg. no. SCZHE-COP-10.

##### Other material.

• 50 ♀♀ and 5 ♂♂, alcohol preserved, same collection data. All specimens were collected from the type locality in August 2013 and September 2023, leg. L. Garlitska.

##### Description of the adult female.

**Figure 2.**
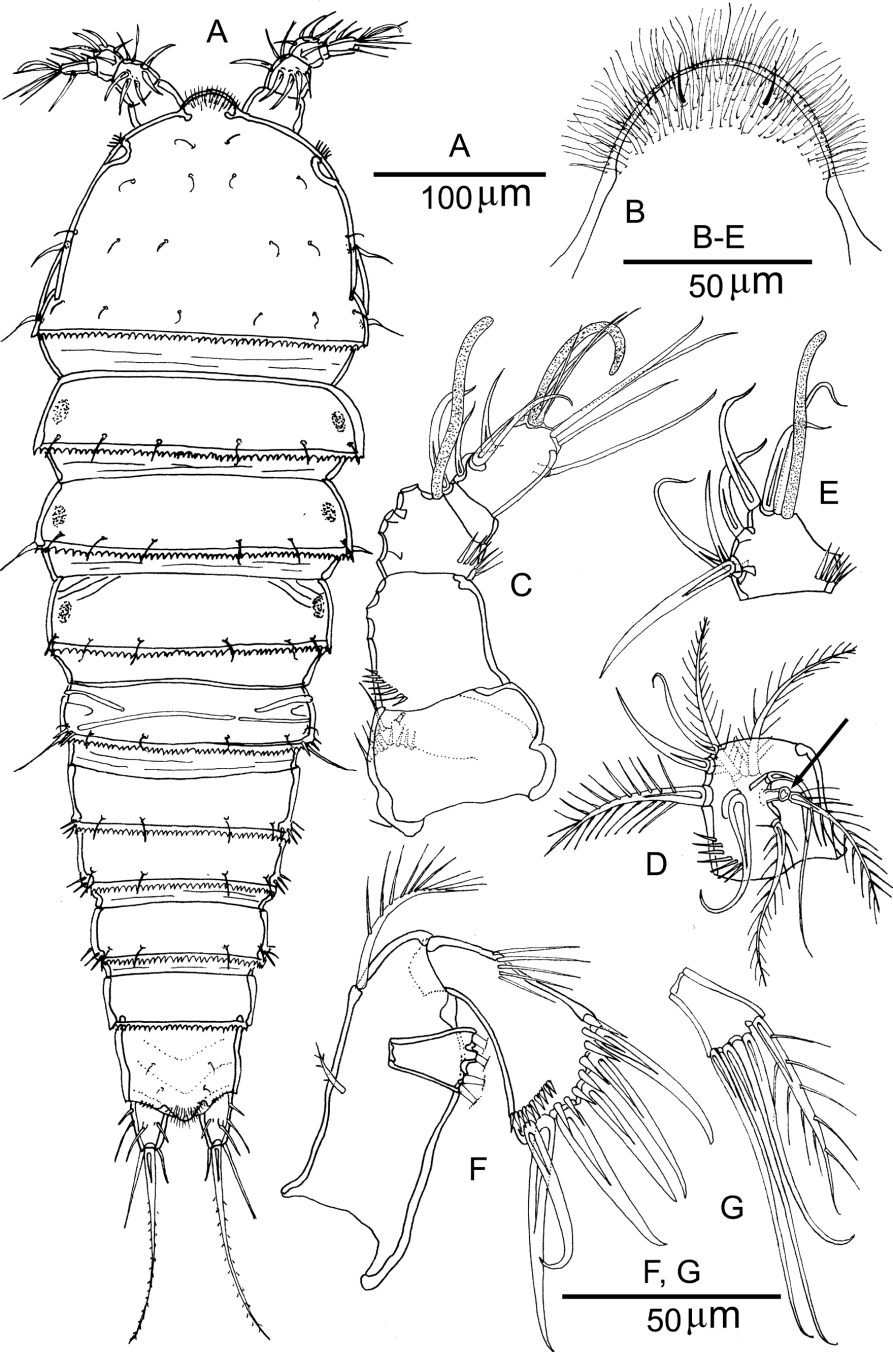
*Nannopus palustris*, female. **A**. Habitus, dorsal; **B**. Rostrum; **C**. Antennule (setae of 2^nd^ and 3^rd^ segments omitted for clarity); **D**. 2^nd^ segment of antennule; **E**. 3^rd^ segment of antennule; **F**. Antenna; **G**. Exopodite of antenna. **B–G**. From neotype.

**Figure 3.**
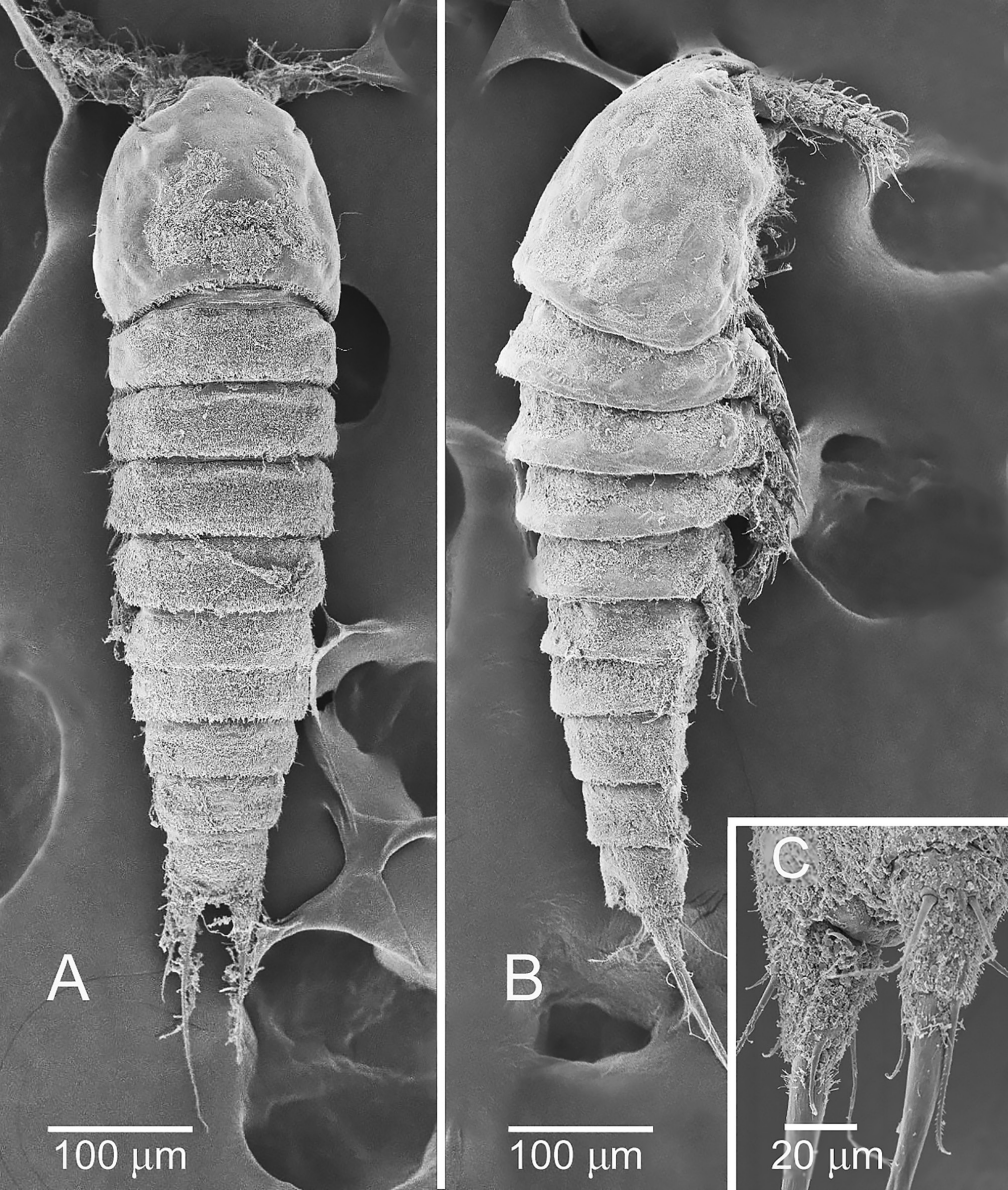
*Nannopus palustris*, female. SEM images showing **A**. Dorsal; **B**. Lateral views; **C**. Caudal rami in ventral view.

**Figure 4.**
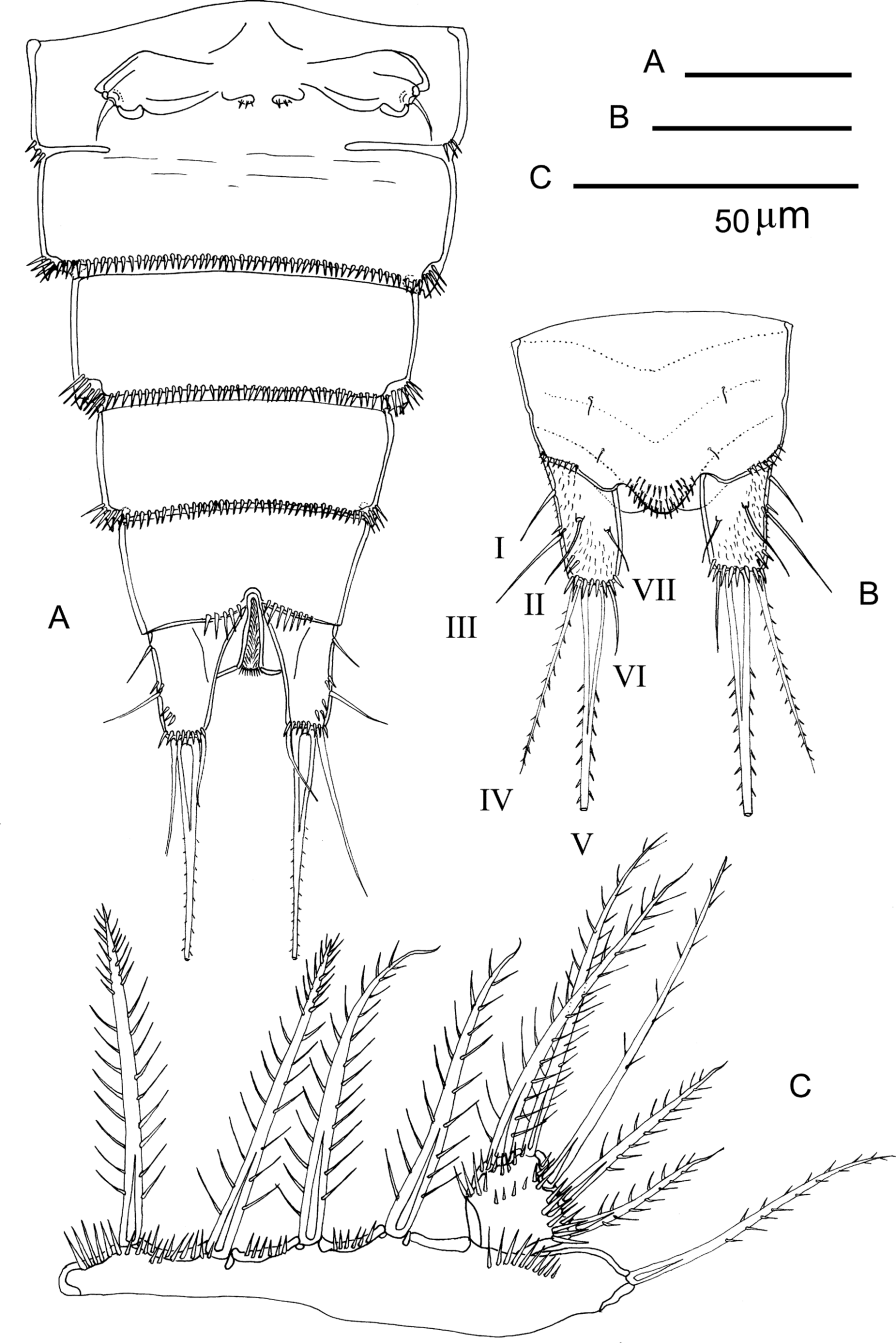
*Nannopus palustris*, female neotype. **A**. Urosome, ventral; **B**. Anal somite with caudal rami, dorsal; **C**. P5.

Total body length measured from the tip of the rostrum to the posterior margin of the caudal rami ranged from 618 to 699 µm (mean = 667 µm; *n* = 10). Habitus fusiform (Figs 2A, 3). Color of preserved specimens pale yellowish to colorless. Rostrum prominent, fused to the cephalothorax, recurved ventrally; anterior margin with multiple long and slender setules and one pair of sensilla (Fig. 2B). Cphth bell-shaped, representing 22% of the total body length; posterior margin serrate. Sensillar pattern on the cephalothorax and body somites are illustrated in Fig. 2A, B. Posterodorsal margin of each somite serrate except for the anal somite. Somites bearing P2–P4 with pairs of lateral nuchal organs (osmoregulatory integumental windows) (Fig. 2A). Genital and first abdominal somite fused, forming a GDS; the original segmentation marked by bilateral constriction and by a transverse serrate ridge dorsally. Anal somite cleft medially in ventral view; ventral posterior margin with row of spinules (Fig. 4A); dorsal surface with two pairs of sensilla (Fig. 4B). Anal operculum well developed, with rows of setules (Fig. 4B).

***Caudal ramus*** (Figs 3C, 4B) cylindrical and elongate, dorsal posterior margin with row of spinules, with seven setae. Seta I naked, inserted close to the anterior margin of the ramus. Seta II naked, located dorsolaterally. Seta III naked, inserted approximately halfway down the outer margin. Seta IV bipinnate, longer than ramus (in dorsal view); located at the outer distal corner. Seta V longest (2.6–3× longer than IV), strongest, and swollen at its base and bipinnate. Seta VI naked; located at inner distal corner. Dorsal seta VII naked; located close to inner margin.

***Antennule*** (Fig. 2C–E) compact, short, and 5-segmented. First, second and third segments with rows of spinules. First segment with one posterior seta and pore. Antennule 2^nd^ segment with a tri-articulate seta inserted dorsally. Segment 3 with aesthetasc fused basally to long seta. Segment 5 with acrothek. Armature as follows: 1-[1], 2-[4 + 5 bipinnate], 3-[6 + (1 + ae)], 4-[1], 5-[8 + acrothek].

***Antenna*** (Fig. 2F, G). Relatively short, consisting of allobasis, a 1-segmented endopod and a 1-segmented exopod. Allobasis with two abexopodal setae of which one short bipinnate on the middle margin, and one long pinnate on the distal margin. Endopod with a transverse row of spinules in the proximal half; a row of robust spinules near the inner corner and a row of short spinules near the outer corner; armed with six strong spines. Exopod with four elements, one of which is bipinnate.

**Figure 5.**
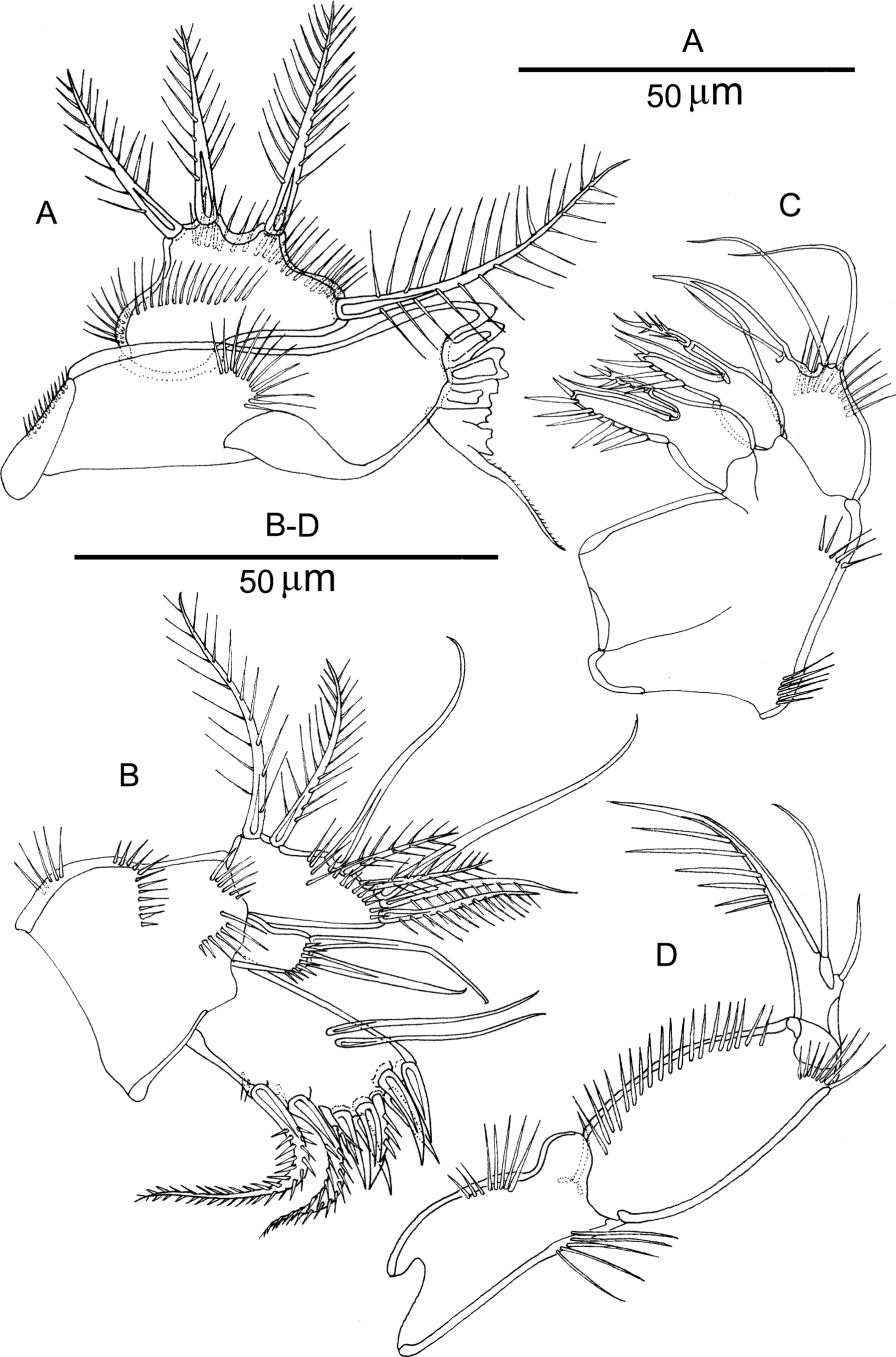
*Nannopus palustris*, female neotype. **A**. Mandible; **B**. Maxillule; **C**. Maxilla; **D**. Maxilliped.

***Mandible*** (Fig. 5A). Gnathobase is well developed; the cutting edge is provided with six rigid, multicuspidate teeth and a setular process. Mandibular palp 1-segmented with rami completely incorporated into the basis; outer margin and middle part with rows of spinules; armed with four bipinnate setae, one of which (derived from the basis) originates from a subcylindrical pedestal.

***Maxillule*** (Fig. 5B). Praecoxal arthrite with two setae on the anterior surface; distal margin with six stout spines, two short pinnate and two long pinnate elements. Syncoxa with cylindrical coxal endite bearing two naked setae. Basis and rami fused; armed with two naked, four bipinnate and two pinnate setae.

***Maxilla*** (Fig. 5C). Syncoxa bearing two subequal endites, each with three elements (2 spinulose which are confluent with endites, and 1 slender naked). Allobasis with a row of spinules, a strong claw with one accompanying seta. Endopod incorporated into basis and represent by two naked setae.

***Maxilliped*** (Fig. 5D) subchelate, 3-segmented. Syncoxa shorter than basis, with rows of spinules, unarmed. Basis unarmed; with rows of spinules. Endopod with a strong, curved claw ornamented with spinules in the distal half, and two naked accessory setae.

**Figure 6.**
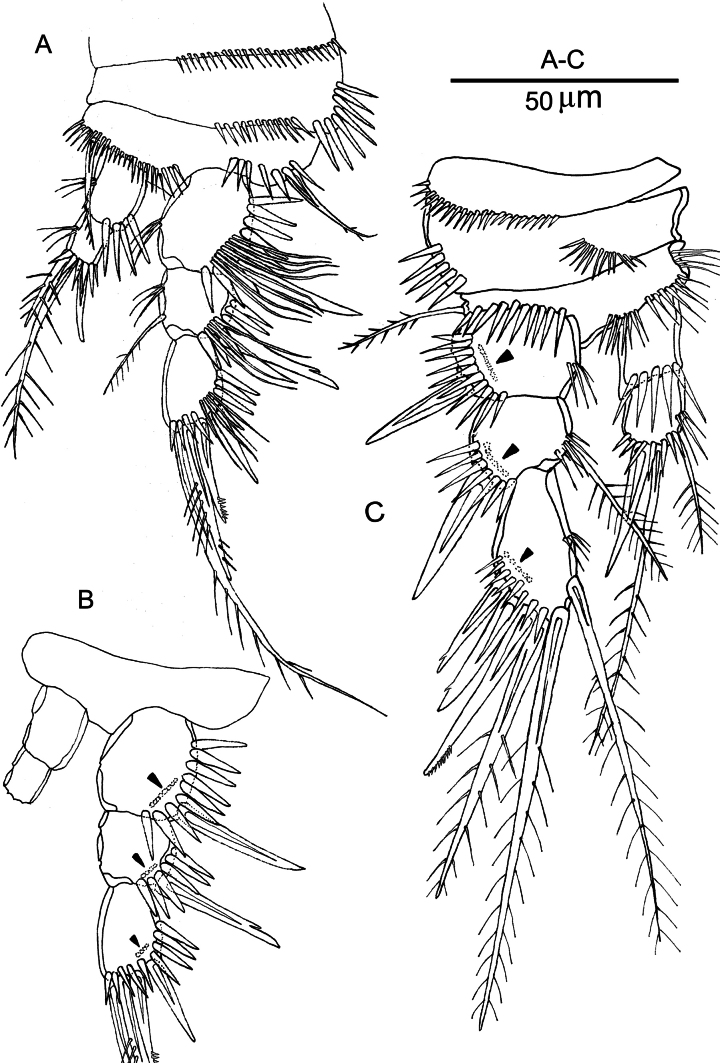
*Nannopus palustris*, female neotype. **A**. P1, anterior; **B**. P1, anterior; **C**. P2, anterior. Arrows indicate where thin setules attached.

**Figure 7.**
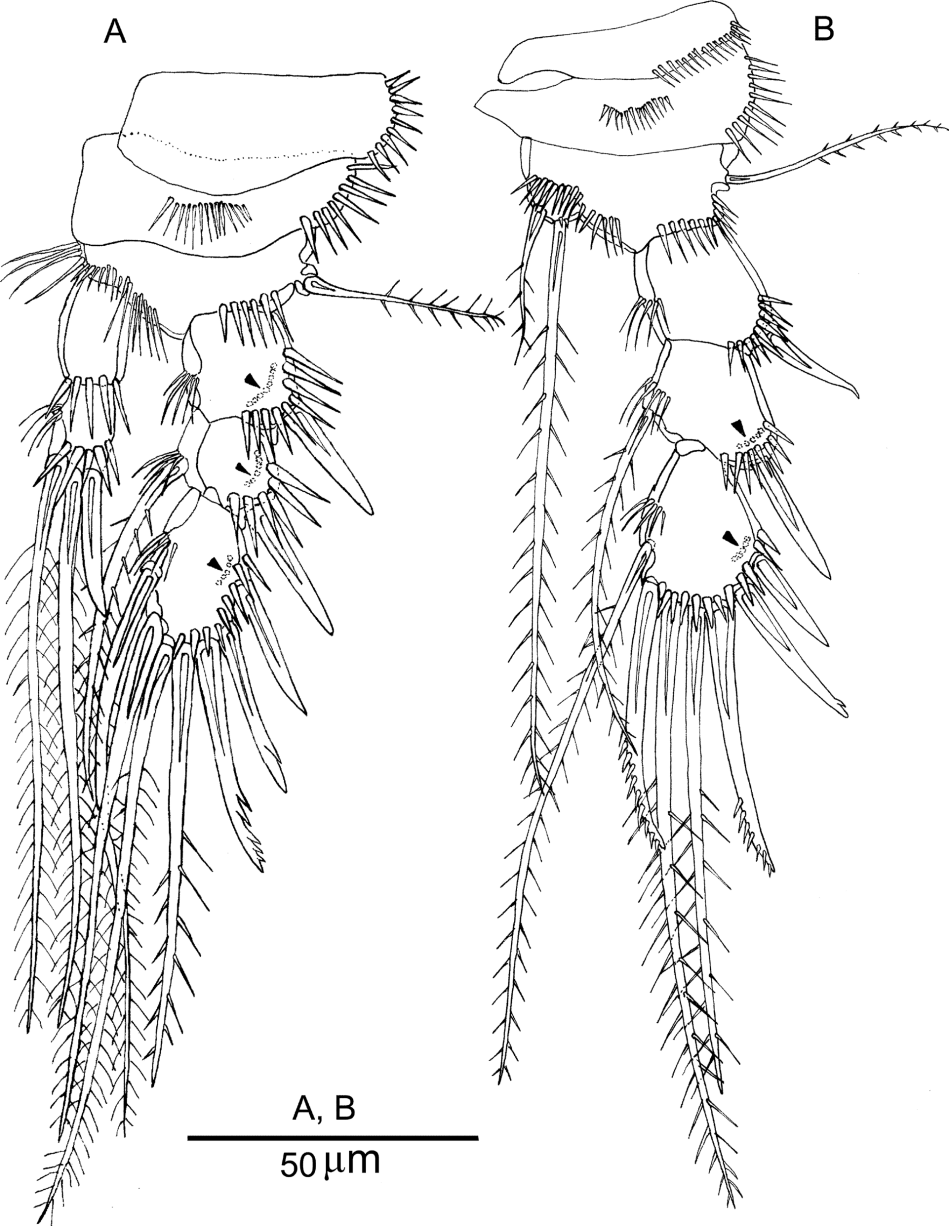
*Nannopus palustris*, female neotype. **A**. P3, anterior; **B**. P4, anterior. Arrows indicate where thin setules attached.

***Swimming legs*** (Figs 6, 7) with 3-segmented exopods and 2- (P1–P3) or 1-segmented (P4) endopods; exopods longer than endopods. P1–P4 with smooth and short concave intercoxal sclerites (not shown). Praecoxae with row of spinules on the anterior surface. Coxae with a row of spinules on the anterior surface and a row of strong spinules along the outer margin. Bases with plumose outer seta; with rows of spinules near the insertion of the exopod and endopod. Exopodal segments (except P1exp-3) with bunches of thin setules on inner margin; first (except P4), second and third segments of the exopod with row of tiny setules near the outer distal corner (Fig. 6A, on Figs 6B, C, 7 marked with arrows); outer margins of exopodal and endopodal segments with spinular ornamentation, except for the P4 endopod.

***P1*** (Fig. 6A, B). Basis with row of spinules near the insertion of the inner and outer spines; inner basal spine pinnate. Outer exopodal spine of exp-1 naked; exp-2 with outer spine with single tooth and with inner seta; exp-3 with one naked and one pectinate outer spine, and two terminal elements of which outer bipinnate and inner plumose. Enp-1 and end-2 with bunches of tiny setules on inner margin; outer element of enp-2 naked, terminal element bipinnate, and inner seta short and pinnate.

***P2*** (Fig. 6C). Basis with a row of long setules along the inner margin. Inner margins of all segments with setular row; outer exopodal spines of exp-1 and exp-2 naked. Exp-3 with three outer spines (of which two proximal ones with a tooth, and distal one pectinate), two apical setae of which outer pinnate and inner plumose, and one inner plumose element. Inner margin of end-2 with setular row, outer element of enp-2 spiniform and naked, terminal and inner elements setiform and plumose.

***P3*** (Fig. 7A). Basis with a row of long setules along the inner margin. Inner margin of all segments (except end-1) with setular row; outer exopodal spines of exp-1 and exp-2 naked. First outer spine of exp-3 naked, second spine with single tooth and distal spine pectinate; inner and terminal elements plumose. Outer element of enp-2 spiniform and naked; terminal and inner element long, setiform and plumose.

***P4*** (Fig. 7B). Inner margin of exopodal segments with row of setules. Outer spine of exp-1 and exp-2 naked; first outer spine of exp-3 naked, second spine with single tooth and distal spine pectinate; inner element of exp-3 plumose seta; inner subdistal seta shortest and pectinate; inner apical elements plumose. Endopod with long plumose apical seta, and a short (~ 4× shorter than apical) plumose inner element.

Setal formula of the swimming legs is as follows:

**Table:** 

	Exp	Enp
P1	0.1.022	0.111
P2	0.1.123	0.111
P3	0.1.223	0.111
P4	0.1.223	110

***P5*** (Fig. 4C). Baseoendopod transversely elongate, with distal spinules along the plate and near articulation with the exopod; endopodal lobe with four bipinnate elements; outer expansion with bipinnate basal seta. Exopod covered with spinules, articulating with baseoendopod; semicircular and approximately as long as wide; with five pinnate elements.

***P6*** (Fig. 4A). Closing off paired genital apertures, semi-triangular with protruding outer distal edge bearing one short naked element.

##### Description of the adult male.

Total body length measured from the tip of the rostrum to the posterior margin of the caudal rami ranged from 563 to 610 µm (mean = 588 µm; *n* = 5). Sexual dimorphism expressed in body size, antennule, P2, P3, P5, P6, and urosomal segmentation.

**Figure 8.**
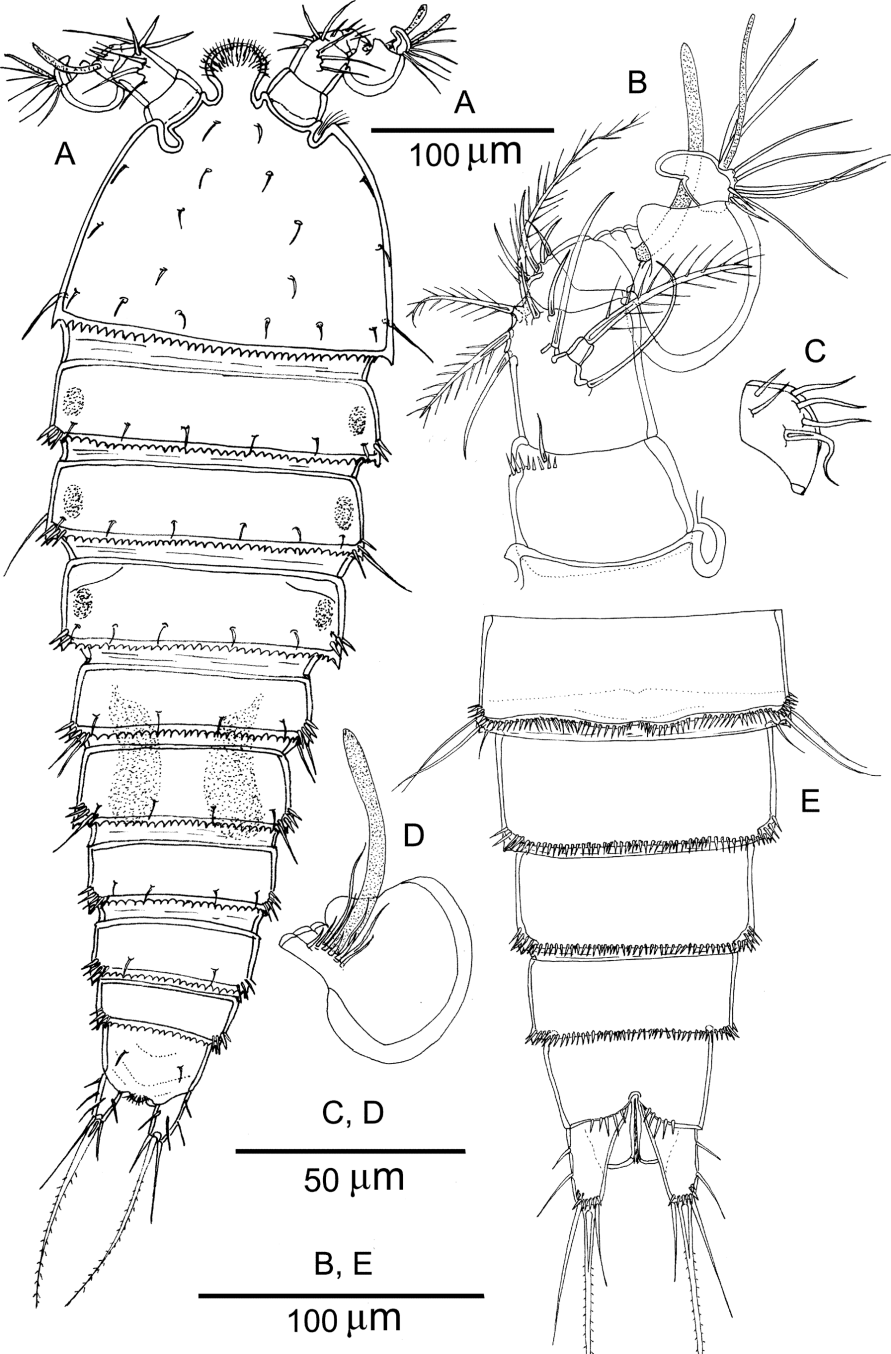
*Nannopus palustris*, male. **A**. Habitus, dorsal; **B**. Antennule; **C**. 4^th^ segment of antennule; **D**. 5^th^ segment of antennule; **E**. Urosome, ventral.

Ornamentation of the body and armature of the caudal ramus like in female (Fig. 8A, E).

***Antennule*** (Fig. 8B–D) short, 6-segmented; geniculation located between segments 4 and 5. All segments are strongly chitinized. First segment with a row of spinules and a small naked seta. Segment 5 with aesthetasc fused basally to long seta. Segment 6 with acrothek. The armature formula is as follows: 1-[1], 2-[5 + 4 bipinnate], 3-[4], 4-[6], 5-[8 + (1 + ae)], 6-[7 + acrothek].

***Antenna***, mouthparts, and P1 like in female.

**Figure 9.**
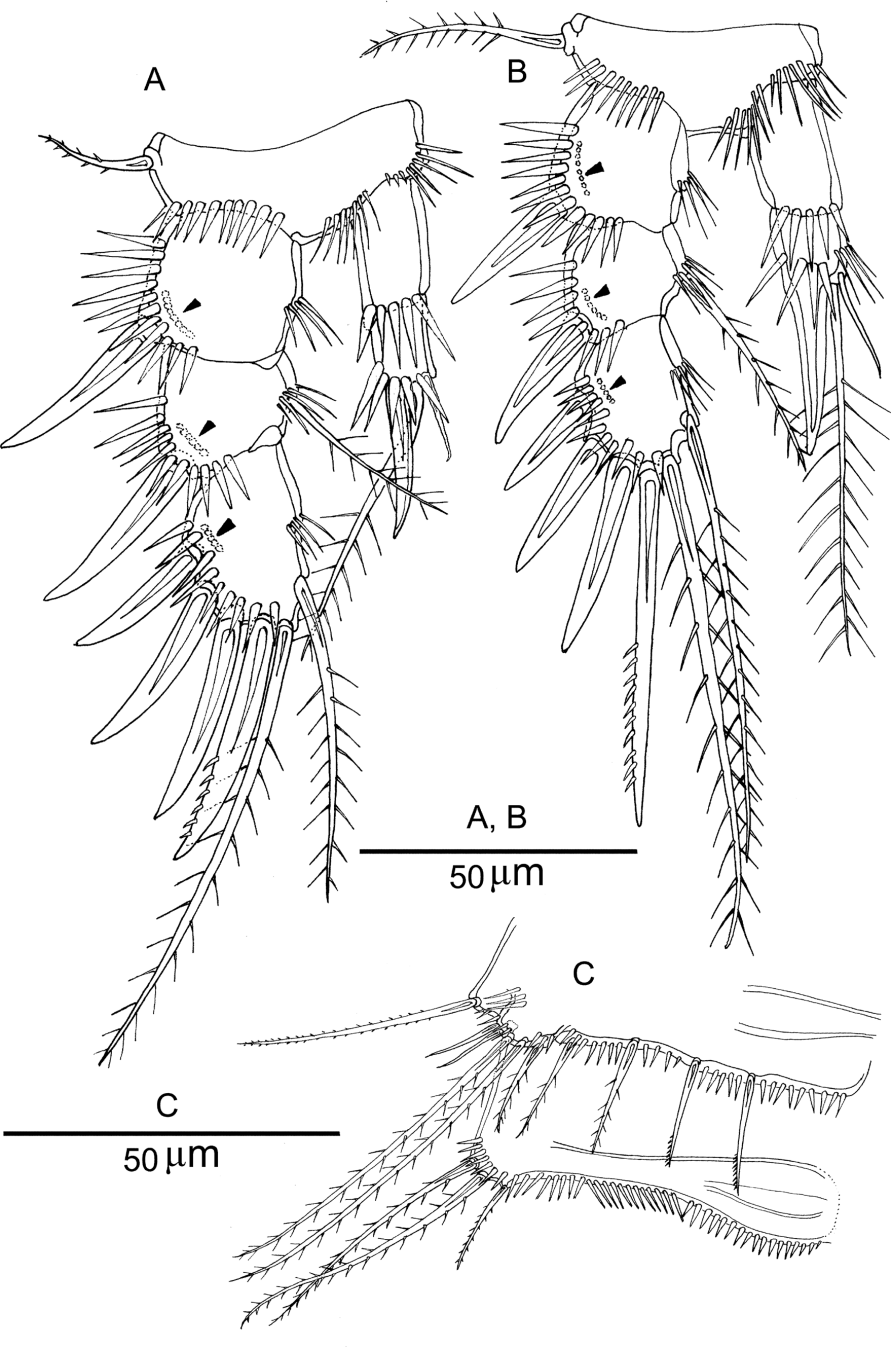
*Nannopus palustris*, male. **A**. P2, anterior; **B**. P3, anterior; **C**. P5 and P6, anterior. Arrows indicate where thin setules attached.

***P2 and P3*** (Fig. 9A, B). Exp-1, -2 and -3 of both legs with outer spines stronger than in female; the outer terminal spine of exp-3 with spinules and stronger than in female. P3enp-2 (Fig. 8B), with the outer spine forming a basally fused, robust apophysis; terminal and inner elements shorter than those in female; inner element naked.

***P5*** (Fig. 9C) fused with somite. Baseoendopod and exopod fused, forming a common plate with a long row of spinules. Endopodal lobe armed with two pectinate and two bipinnate elements. Outer setophore with bipinnate basal seta. Exopodal lobe with three plumose and two naked elements.

***P6*** (Fig. 9C) asymmetrical with a functional left leg forming a simple flap; confluent with somite, with spinules along the distal margin and armed with one bipinnate and two plumose elements. Double spermatophore observed underneath the flap (not shown).

##### Additional comments.

The body and appendages of the specimens were densely covered with mud, which could be only partly removed by ultrasonication. The natural population in the type locality exhibited a rather unbalanced sex ratio (in August 2013 and September 2023, females accounted for 90–95% of adults).

### Molecular and phylogenetic analyses

**Figure 10.**
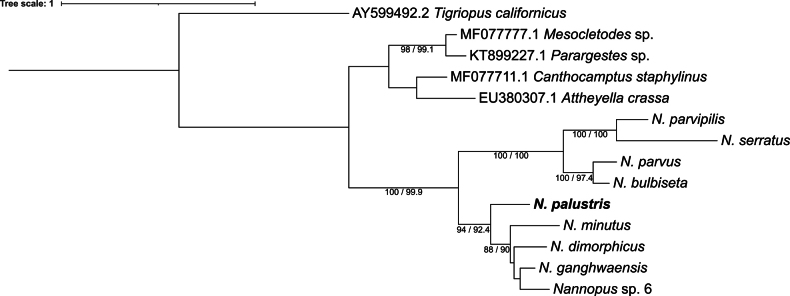
ML phylogenetic tree based on concatenated 18S and COI sequences of *Nannopus*. Branch supports values at nodes are given as bootstrap/SH-aLRT percentages. *Nannopus palustris*, for which sequences were obtained in the present study, is highlighted in bold.

COI and 18S sequences obtained for three specimens of *N. palustris* were identical (Table 2). The ML phylogenetic analysis of the data set of nine species of *Nannopus* based on 18S and COI sequences is retained as monophyletic, with two highly divergent clades supported with strong bootstrap and SH-aLRT values (100 and 99.9%), indicating the presence of two divergent lineages (Fig. 10). The first clade included *N. parvus*, *N. bulbiseta*, *N. parvipilis*, and *N. serratus*, with clear interspecific divergences within the clade (BP = 100%). The second clade included *N. palustris*, *N. minutus*, *N. dimorphicus*, *N. ganghwaensis*, and *Nannopus* sp. MK075977.1 (sp6, the member of *N. ganghwaensis* cryptic complex, in Vakati et al. 2019). However, within the second clade *N. palustris* formed a single distinct lineage with relatively strong support (94 and 92.4%). Genetic distances (uncorrected *p*-distances) for COI between *N. palustris* and other species from the second clade ranged from 23.9 to 25.9%.

## Discussion

**Figure 11.**
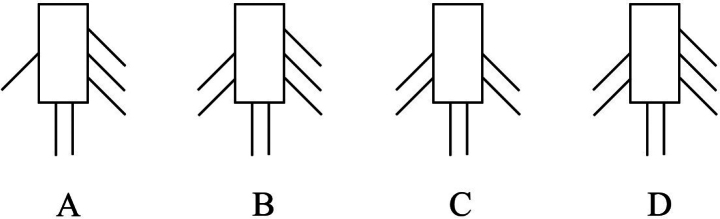
*N. palustris*, P4exp-3 (left side – inner margin, right side – outer margin). **A**. Brady 1880; **B**. T. Scott 1902; **C**. Sciberras et al. 2021; **D**. Our specimens from the type locality.

As mentioned earlier, in 1880, G. Stewardson Brady briefly described a harpacticoid from Seaton Sluice (Great Britain) and named it *Nannopus palustris*. In 1892, Eugène Canu found similar harpacticoids (females only) in the La Manche region, France, and assigned them to the same species. These specimens, however, were different in the number of endopod segments, as well as in the setal formula. Canu (1892) suggested that P1 in Brady's drawings were nothing more than P2 or P3. Later, many authors (e.g., Fiers and Kotwicki 2013; Kim et al. 2017) expressed doubts about the identity of *Nannopus palustris* sensu Canu (1892). Sciberras et al. (2021) designated the female specimen illustrated by Canu (1892: pl. IV, figs 6–21) as the holotype of *Nannopus canui* Sciberras, Cazzaniga & Huys, 2021.

In the summer of 1901, Thomas Scott (1902) found palustris-like harpacticoids near Newburgh on the Ythan, Aberdeenshire (Scotland), in a habitat similar to that studied by Brady. There are, however, a few dissimilarities between his drawing and our specimens. Specifically, these include the following: 1) the absence of one seta on the maxilliped claw and 2) an extra inner seta on P2exp-3 (we agree with Fiers and Kotwicki (2013) that this was in fact the female P3). If we disregard these minor errors in Scott’s drawings and consider the proximity of the location to the type locality, we believe that he found and described *N. palustris*. Willey (1929) reported males with two symmetrically located spermatophores. Vakati et al. (2019) reported that only *N. didelphis* and *N. procerus* have two spermatophores. Fiers and Kotwicki (2013) assumed some similarity between Willey’s (1929)*N. palustris* and *N. procerus*.

According to Sciberras et al. (2021: 520), P4exp-3 of *N. palustris* has an armature formula of 222 (Fig. 11C). However, Brady’s original drawing (1880: pl. 77; fig. 19) shows a setal formula of 123 (Fig. 11A), which also occurs in *N. scaldicola* and *N. procerus* (Fiers and Kotwicki 2013). Scott’s drawing (1902: pl. 23; fig. 21) indicates setation 223 (Fig. 11B). Our material from the Seaton Sluice also has the P4exp-3 pattern 223 (Figs 7B, 11D), and the setal ornamentation is in accordance with Scott’s figure. We suppose that Brady’s drawing was not correct, especially since he wrote:

“I have seen only one or two specimens of this species, which were so coated with mud that it was difficult to get a distinct view of the limbs, nor did I at first recognise anything very noteworthy about them, else possibly a little extra care might have enabled me more fully to elucidate their structure” (Brady 1880: 101).

### Corroboration for *Nannopus* subdivision and position of *N. palustris*

Sciberras et al. (2021) subdivided the genus into two species groups primarily on the basis of the presence or absence of the inner seta on P1exp-2. They marked this character state as unknown for *N. palustris*, referring to Brady’s (1880: fig. 19) confusing original illustration. This seta is present in our specimens (see above). Moreover, the armature formulae of P1–P4 displayed by the females of our *N. palustris* also corresponds exactly to those for most members of Group II (Sciberras et al. 2021: Table 2); thus, the type species, *N. palustris*, can be assigned with certainty to this group. The key characters of *N. palustris* according the topotype specimens are summarized in Table 3. Therefore, it was misleading to include *N. palustris* basing on Brady’s description in the key to the valid species of *Nannopus* (Vakati and Lee 2021: couplets #1, 2).

**Table 3.** Morphological characters of *Nannopus palustris* Brady, 1880. N: naked seta; P: pinnate seta.

Characters	
Shape of habitus (dorsally) ♀ and ♂	Fusiform and broad at posterior region of cephalothorax
Body length ♀: ♂ (in µm)	667: 588
A1 no. of segments ♀	5
A1 armature formula ♀	1–[1], 2–[4 + 5 bipinnate], 3–[6 + (1 + ae)], 4–[1], 5–[8 + (2 + acrothek)]
A2 exopodal setae ♀ and ♂	4 (3N + 1P)
A2 endopodal spines ♀ and ♂	6 (unmodified)
A2 abexopodal seta ♀ and ♂	2 (P)
Mandibular palp total no. of elements ♀ and ♂	4 (P)
Maxillule praecoxal arthrite no. of elements at distal margin ♀ and ♂	10 (6 short smooth stout + 2 short stout pinnate + 2 long recurved bipinnate elements)
Integumental windows on cephalothorax ♀	On cephalothorax absent; 3 pairs on P2–P4 somites
P1–P4 endopod segments ♀ and ♂	2:2:2:1-segmented
P1exp-2 innermost seta	Present
P2enp-2 innermost seta ♀	Almost as long as the outer spine of its segment
P2–P3exp-3 outer spines ♀ and ♂	3-3: 3-3
P3enp-2 ♂	Rectangular, and distal spine fused forming sharp apophysis
P4exp-3 inner subdistal element ♀ and ♂	Pectinate seta
P5 exopod articulation to baseoendopod ♀	Not fused
P6 articulation ♂	Asymmetrically confluent
Caudal rami seta IV ♀: ♂	Inflated
Caudal rami setae V ♀: ♂	Inflated
Caudal rami setae ornamentation I:II:III:IV:V:VI:VII ♀	N: N: N: P: P: N: N
Shape of caudal rami ♀ and ♂	Cylindrical
Caudal rami ventrally♀ and ♂	Half the rami are outside the anal somite

The molecular data obtained by Vakati et al. (2019) using mitochondrial and nuclear genes for several species from Korean coasts generally supported this subdivision, indicating two clearly separated clades. Specifically, the first included four species assigned by Sciberras et al. (2021) to Group I, and the second included three species from Group II (and two yet undescribed cryptic species from the *N. ganghwaensis* species complex). Our 18S rDNA-COI-based ML tree was also divided into two major clades with strong support; both corresponding well to those obtained by Vakati et al. (2019). Furthermore, with certainty, *N. palustris* belonged to Group II but was distinctly separated from the other members of the group (Fig. 10). Thus, both molecular and morphological data corroborate the proposed subdivision of the genus and support the monophyly of the two groups.

### Comparison with other congeners

*Nannopus palustris* can be clearly distinguished from the members of Group I (*N. parvus*, *N. bulbiseta*, *N. perplexus*, *N. serratus*, *N. robustus*, *N. parvipilis*, and *N. unisegmentatus*) by the swimming leg armature formulae, particularly for P1–P4 exp (Sciberras et al. 2021: Table 2). *Nannopus palustris* can be differentiated from the other members of Group II by the characters below.

*Nannopus ganghwaensis* can be differentiated from *N. palustris* by its larger body size (772:667 vs 667:588 µm for ♀:♂, respectively), ♀ P2enp-2 innermost seta (1.7× longer than the outer spine of its segment vs almost equal in length) and the number of P2–P3exp-3 outer spines in males (4-4 vs 3-3).

*Nannopus dimorphicus* differs from *N. palustris* in terms of shorter body length (408:377 vs 667:588 µm for ♀:♂, respectively), number of P2–P3exp-3 outer spines in males (4-4 vs 3-3), form and ornamentation of caudal rami setae (III ♀: pinnate vs naked; IV: globular at its insertion site vs slender along the full length; V♂: unmodiﬁed vs inflated), and presence of one integumental window on the cephalothorax.

*Nannopus minutus* differs in the shape of its habitus (narrow vs broad at the posterior region of cephalothorax), body length (500:431 vs 667:588 µm for ♀:♂, respectively), form and ornamentation of caudal rami setae IV (slightly bulbous vs slender along the full length) and V (unmodiﬁed and > 5× as long as seta IV vs inflated and 2.5–3× as long as seta IV). Females of *N. minutus* can be differentiated from those of *N. palustris* by the presence of three integumental windows on the cephalothorax (absent in the ♂ and in both sexes of the latter species), ornamentation of caudal rami seta III (pinnate vs naked), and the inner seta of P3enp-2 being distinctly shorter than the apical one (vs both setae being equally long in *N. palustris*).

*Nannopus cylindricus* females (♂ unknown) differ in the conspicuously cylindrical shape of the base of the caudal seta V and P2enp-2 innermost seta (1.7× longer than the outer spine of its segment vs almost equal in length).

*Nannopus scaldicola* differs from *N. palustris* in shape (ovate vs fusiform), shorter body (460–515:460 vs 667:588 µm for ♀:♂, respectively), form of caudal rami setae IV and V (unmodified vs inflated), ornamentation of caudal rami seta VI (pinnate vs naked), presence of one integumental window on ♀ cephalothorax, P2enp-2 innermost seta ♀ (2.6× as long as the outer spine of its segment vs almost equal in length), number of P2–P3exp-3 outer spines in males (4-4 vs 3-3), and the armature of P4exp-3 (single inner subdistal setiform element vs two pectinate setae; and the outer subdistal element pinnate vs stout pectinate one).

*Nannopus procerus* has a slender, slightly fusiform cylindrical body with a nearly parallel-sided metasome (vs markedly fusiform and broad at the posterior region of the cephalothorax), possesses an unmodiﬁed (vs bipinnate inflated), long (4× as long as seta IV) caudal rami seta IV, an anteriorly swollen and notched seta V with a spur at the caudal end (vs shorter, bipinnate inflated seta without spur), P2enp-2 innermost seta twice as long as the outer spine of its segment (vs almost equal in length), and P3 and P4exp-3 have a single inner subdistal spiniform element each (vs two pectinate setae at each segment). Males possess symmetrical, midventrally confluent P6 articulation (vs asymmetrical with only one functional P6).

*Nannopus flexibilis* differs from *N. palustris* in caudal seta IV in females (small naked vs well-developed pinnate), the presence of an integumental window on cephalothorax ♀, the shape of male P3enp-2 (square or globular vs rectangular).

*Nannopus sinusalbi* differs in the number of elements at the distal margin of maxillule praecoxal arthrite (5 stout spines + 2 slender elements vs 6 short smooth stout + 2 short stout pinnate + 2 long recurved bipinnate elements in *N. palustris*), caudal rami setae size and ornamentation (seta III pinnate vs naked, seta IV naked and not basally dilated, almost equal seta VI in length, vs bipinnate, inflated, ~ 3× as long as VI, seta V very long (7× as long as seta IV vs 2.5–3× as long as seta IV), seta VII pinnate vs naked), P2enp-2 innermost seta (pinnate in both sexes vs naked in males), P2exp-2 innermost seta is one-third longer than the outer spine of its segment vs subequal in length.

*Nannopus didelphis* differs from *N. palustris* in the form of caudal rami seta IV (normal unmodiﬁed vs inflated bipinnate) and the ornamentation of elements at the distal margin of the maxillule praecoxal arthrite (8 smooth and stout spines + 2 long recurved pinnate elements vs 6 short smooth stout + 2 short stout pinnate + 2 long recurved bipinnate elements). Additionally, *N. didelphis* males differ in the number of P2–P3exp-3 outer spines (4-4 vs 3-3) and P6 articulation (symmetrically confluent vs asymmetrical with only one functional P6).

*Nannopus hirsutus* females (♂ unknown) differ from *N. palustris* in conspicuous body ornamentation, particularly the setular clusters on the pleurotergites of the urosomites and the outer margins of the caudal rami, in the form of caudal rami setae IV (globular at its insertion site) and V (long cylindrical and heavily ornamented at the anterior region), and the ornamentation of elements at the distal margin of the maxillule praecoxal arthrite (similar to *N. didelphis*).

*Nannopus canui* (with respect to the specimens described by Canu (1892) from the Wimereux mud ﬂats, France), the substantive differences from other descriptions allowed several authors to attribute its distinct specific status (e.g., Fiers and Kotwicki 2013; Kim et al. 2017); it was subsequently attributed as *N. canui* (Sciberras et al. 2021). The species can be differentiated from *N. palustris* s. str. by the following ♀ characters (♂ unknown): slenderer and less ﬂattened fusiform body shape, larger body size (870 vs 667 µm), the number and position of the lateral setae on the caudal rami, the long outer seta IV on the caudal rami (twice as long as the ramus), the form of the subdistal inner element on the P2 endopodite (long setiform element vs the seta a bit shorter than the outer spine of its segment), and the absence of a pectinate distal inner seta on P4exp-3.

### Other “*N. palustris*” records: a brief overview

Harpacticoids originally identified as *N. palustris* have been repeatedly reported from different marine, estuarine and freshwater habitats around the world (see distribution map in Garlitska et al. 2012). Most of these records lacked clear confirmation or, at best, were accompanied by brief and incomplete descriptions (reviewed by Garlitska et al. 2012; Fiers and Kotwicki 2013; Sciberras et al. 2021). Here, we briefly comment on some selected findings, mainly those supported by our own material (see “Additional material” subsection).

Specimens from Westerschelde estuary (SW Netherlands) and Small Adzhalyk Estuary (NW Black Sea) appeared morphologically identical to those from the type locality in most taxonomically significant characters. These populations were slightly smaller than the specimens from the type locality (Netherlands: ♀ 647 ± 73 µm, ♂ 576 ± 29; Black Sea: ♀ 602 ± 30, ♂ 566 ± 11); the only significant difference was for the Black Sea females (Mann-Whitney test: *p* = 0.02). Earlier, these two populations were found to be almost genetically identical based on both mitochondrial (Cyt*b*) and nuclear (28S rDNA) genes, despite their geographical isolation (Garlitska et al. 2012). Therefore, we provisionally attributed these distant populations to *N. palustris*; however, a conclusive decision requires more careful inspection with joint genetic analysis.

Reexamination of the White Sea specimens recognized earlier as *N. palustris* clearly revealed their strong resemblance to *N. procerus*, so we confirm Fiers and Kotwicki’s (2013) assumption that “*N. palustris*” populations reported from the White (Chislenko 1967; Kornev and Chertoprud 2008; Garlitska et al. 2012) and Barents (Letova 1982) seas are conspeciﬁc with *N. procerus*.

Wells (1971) found several specimens (females only) at the intertidal beach in the Vellar estuary, Parangipettai (Bay of Bengal). He assigned these specimens to *N. palustris* but noted some differences from the descriptions of Canu (1892) and T. Scott (1902) in the setation of the P5 exopod, treating this difference as intraspecific variation. In 2012, we collected several *Nannopus* specimens (of both sexes) from the same locality. Inspection of these specimens confirmed the observations of Wells (1971) and revealed several other distinctions between the Vellar and Northumberland populations, namely, i) the absence of the inner seta on P1exp-2 (present in *N. palustris*); ii) P2–P3exp-3 outer spines ♀ and ♂ were 2-2:2-2 (as opposed to 3-3:3-3 for *N. palustris*); iii) P3enp-2 ♂ differed in form; and iv) P5 ♀ baseoendopod and exopod setae were 3:4 (as compared with 4:5). Therefore, the Vellar population possesses a unique combination of characters and is clearly not assignable to *N. palustris* or to any other congener described thus far. Thus, it should be considered a new species, presumably a member of Group I.

With respect to the specimens from Lake Hinuma (Japan) recognized as *N. palustris* by Kikuchi and Yokota (1984), neither male nor female had inner subdistal pectinate seta on P4exp-3. They also differed from *N. palustris* in the form of apophysis of P3 end-2 and in body size (630–700 and 400 µm for ♀ and ♂). Therefore, they most likely belong to a different species.

A number of questionable findings and nominate species of *Nannopus* reported worldwide still await re-evaluation, morphological revision, and/or genetic analyses. This further work will likely require updates and adjustments to the recently published identification keys for the genus (Vakati et al. 2016; Vakati and Lee 2017, 2021; Sciberras et al. 2021). Before these revisions are complete, we refrain from providing a new identification key.

## Conclusions

Our study provides a detailed redescription of *N. palustris* s. str. on the basis of specimens from the type locality. We have specified the key distinguishing features for both sexes of the species and evaluated their phylogenetic relationships using 18S rDNA nuclear gene sequences. Considering both the morphological traits and genetic evidence, we conclude that *N. palustris* is a distinct species within Group II of the genus. Furthermore, our examination of several other geographically distant populations suggests that some may represent different species. Owing to recent findings, *Nannopus* is recognized as a rather diverse genus, containing several complexes of cryptic or pseudocryptic species that can be easily misidentified (Staton et al. 2005; Garlitska et al. 2012; Vakati et al. 2019). Our findings help resolve ambiguities and disagreements in earlier descriptions of this enigmatic taxon and provide a taxonomic framework for related species delimitation within the genus. However, further comparative morphological and molecular studies are needed to untangle the complex phylogenetic relationships of *Nannopus* and avoid any further taxonomic confusion.

## Supplementary Material

XML Treatment for Nannopus

XML Treatment for Nannopus palustris

## Additional information

### Artificial Intelligence (AI) use

The authors accept full responsibility for the content of the manuscript, including the disclosure of any use of AI.

No AI tools were used in the preparation of this manuscript.

### Author ORCIDs

L. Garlitska https://orcid.org/0000-0002-1317-729X

E. Chertoprud https://orcid.org/0000-0002-9874-1610

T. Mayor https://orcid.org/0000-0003-4425-1330

M. Saburova https://orcid.org/0000-0002-2204-2485

G. Gevorgyan https://orcid.org/0000-0002-3450-4405

A. Azovsky https://orcid.org/0000-0001-6815-3968

### Data availability

The 18S rRNA and COI gene sequences of *N. palustris* have been deposited in the GenBank under accession numbers PX936547, PX936548, PX980509, PX936533–PX936535.
